# Hyperferritinemia Is Associated with Higher Adiposity, Metabolic Syndrome, and Hepatic Dysfunction, Mainly Affecting Men: A Study in Southern Brazil

**DOI:** 10.3390/pathophysiology32040064

**Published:** 2025-11-19

**Authors:** Késia Zanuzo, Márcia Fernandes Nishiyama, Eloá Angélica Koehnlein, Sabrina Grassiolli

**Affiliations:** 1 Postgraduate Program in Biosciences and Health, State University of Western Paraná (UNIOESTE), Cascavel 85819-110, Brazil; kesiazanuzo@gmail.com; 2 Campus Realeza, Federal University of Fronteira Sul (UFFS), Realeza 85770-000, Brazil; marcia.nishiyama@uffs.edu.br; 3 Postgraduate Program in Food Science and Technology and Nutrition Course, Federal University of Fronteira Sul (UFFS), Realeza 85770-000, Brazil; eloa.koehnlein@uffs.edu.br

**Keywords:** adiposity, insulin resistance, iron

## Abstract

**Objectives**: Serum ferritin (SF) reflects iron homeostasis, in addition to being an acute phase reactant protein. Since its levels are altered in the obesity state, we compared body composition, metabolic profile, liver alterations, and dietary patterns in adults stratified by SF levels (normal vs. high). **Methods**: A cross-sectional study was conducted using secondary data from 113 adults (≥18 years) of both sexes, attended at an outpatient nutrition clinic in southern Brazil and categorized for normal or high SF. Socioeconomic, anthropometric, blood pressure, dietary, biochemical, and liver parameters were assessed and statistical analyses performed. **Results**: Participants with high SF were more frequently male (*p* < 0.0001), married or in a civil union (*p* = 0.012), and had lower educational levels (*p* = 0.009). Moreover, higher rates of obesity (*p* = 0.003), cardiovascular risk (*p* = 0.004), increased body fat percentage (BF%; *p* = 0.002) and metabolic disturbances such as elevated glucose (*p* = 0.023), triglycerides (*p* = 0.003), insulin resistance (*p* = 0.027), hypertension (*p* = 0.001), and metabolic syndrome (MS) (*p* = 0.001) were noted in this group. Liver-related findings comprised increased ALT (*p* = 0.008), uric acid (*p* = 0.016), and indicators of steatosis (*p* = 0.022). Logistic regression demonstrated a higher likelihood of elevated SF among men (OR = 16.82) and individuals with increased BF% (OR = 7.5), without significant influence of diet. **Conclusions**: Adults with elevated SF were predominantly obese men with excess adiposity, insulin resistance, and metabolic and hepatic dysfunctions, conditions that increase the risk of MS and liver injury. These findings suggest that SF and other iron biomarkers may serve as valuable tools for diagnosing metabolic dysfunctions and obesity-related liver diseases, particularly Metabolic Dysfunction-Associated Steatotic Liver Disease (MASLD).

## 1. Introduction

Iron is essential for fundamental physiological processes, including oxygen transport, mitochondrial ATP production, enzymatic activity, and redox balance [1]. Maintaining iron homeostasis is therefore critical for health, as disturbances are frequently associated with conditions such as anemia, inflammation, diabetes, and obesity [2,3]. Ferritin, a protein with ferroxidase activity, plays a central role in this regulation by controlling intracellular iron availability. Its heavy-chain subunits oxidize ferrous iron (Fe^2+^) to the less reactive ferric state (Fe^3+^), enabling safe storage in a bioavailable form and preventing oxidative damage [4].

Alterations in serum ferritin (SF) levels are associated with multiple health outcomes [5], and SF is widely used as a biomarker of body iron stores [6,7]. Low SF concentrations typically indicate iron deficiency, whereas elevated SF suggests pathological iron overload [8]. The exact origin of circulating SF is not fully understood, although macrophages are considered the primary source of systemic ferritin [9,10]. In addition, SF is recognized as an acute-phase reactant, as its levels increase during inflammation, independently of intracellular iron storage [11].

Overweight and obesity are major risk factors for the development of chronic non-communicable diseases (NCDs), particularly type 2 diabetes mellitus (T2DM) and cardiovascular diseases (CVDs) [12,13]. Interestingly, disturbances in iron metabolism, especially elevated SF, are frequently observed in individuals with T2DM, hypertension, dyslipidemia, increased fasting insulin and glucose levels, and central adiposity [14].

Excessive white adipose tissue (WAT) deposition is a hallmark of obesity, and the immuno-metabolic interface plays a key role in understanding the pathological states associated with obesity and in guiding appropriate treatment strategies [15]. During WAT expansion, particularly in visceral depots, a pro-inflammatory state develops, characterized by increased local (intra-organ) and circulating levels of tumor necrosis factor alpha (TNF-α) and interleukins 1 (IL-1) and 6 (IL-6), which are major contributors to insulin resistance (IR) [16]. Greater visceral adiposity, IR, dyslipidemia, glucose intolerance, and hypertension are common metabolic disturbances that cluster within metabolic syndrome (MS), a recognized risk factor for T2DM, as well as cardiovascular and hepatic diseases [14,17].

The liver plays a central role in iron homeostasis, immune responsiveness, and overall metabolism [18]. Elevated IL-6 stimulates hepatic hepcidin production, which inhibits ferroportin activity, thereby blocking intestinal iron absorption and preventing iron efflux from intracellular depots [1]. Consequently, in obesity and MS, iron overload may occur in several tissues, exceeding ferritin’s storage capacity and resulting in hyperferritinemia [19,20]. Excessive hepatic iron retention enhances reactive oxygen species (ROS) generation, promoting a pro-inflammatory state and hepatic insulin resistance, with deleterious effects on lipid and glucose homeostasis [17,21]. Furthermore, the role of inflammation has not been systematically considered in this context, contributing to conflicting findings in the literature [22,23].

Metabolic Dysfunction-Associated Steatotic Liver Disease (MASLD) is closely linked to obesity and IR, particularly when associated with MS. Iron overload is commonly observed in MASLD and is frequently associated with an increased risk of progression to metabolic dysfunction-associated steatohepatitis (MASH), a more severe manifestation of MASLD, as well as to fibrosis, cirrhosis, MASH-related hepatocellular carcinoma (HCC), and overall liver-related mortality [24]. Moreover, hepatic iron accumulation can trigger ferroptosis, a recently described form of regulated cell death driven by excessive ROS production and lipid peroxidation [25]. Ferroptosis also reduces insulin secretion by pancreatic β-cells, thereby exacerbating metabolic dysfunction [7,26]. Additionally, experimental models suggest that insulin induces ferritin synthesis at the mRNA level, providing a potential explanation for the hyperferritinemia frequently observed in the context of IR [5]. Therefore, evaluating SF levels and their association with MS is critical for preventing MASH and its deleterious hepatic consequences.

Several studies have suggested that SF may serve as a non-invasive biomarker for assessing different stages of MASLD [11]. However, systematic reviews [27,28] have reported inconsistent associations between SF and MASLD, indicating that further research is needed to establish the definitive clinical utility of SF in this context. Moreover, sex differences influence iron homeostasis, including SF levels, with men generally presenting higher values than women due to variations in iron storage and regulation [5]. In this context, the present study aimed to verify the association between SF levels (normal vs. high), in both sexes, and its relationship with body composition, metabolic status, dietary patterns and liver dysfunction.

## 2. Materials and Methods

The present study was a cross-sectional, quantitative investigation based on data collected from nutritional assessments performed during the initial consultation of adult individuals (≥18 years) of both sexes who attended an outpatient nutrition clinic in southern Brazil between March 2023 and October 2024. The study protocol was approved by the Research Ethics Committee of the Federal University of the Southern Frontier (UFFS; approval number 41154814.7.0000.5564). All participants provided written informed consent for the use of their socioeconomic, lifestyle, anthropometric, dietary, and metabolic data. The outpatient nutrition clinic offers free public services, and laboratory tests were performed within 30 days of the first consultation by the municipal laboratory, which also provided these tests at no cost. The study was conducted in accordance with the Declarations of Helsinki and Istanbul.

**Figure 1:**
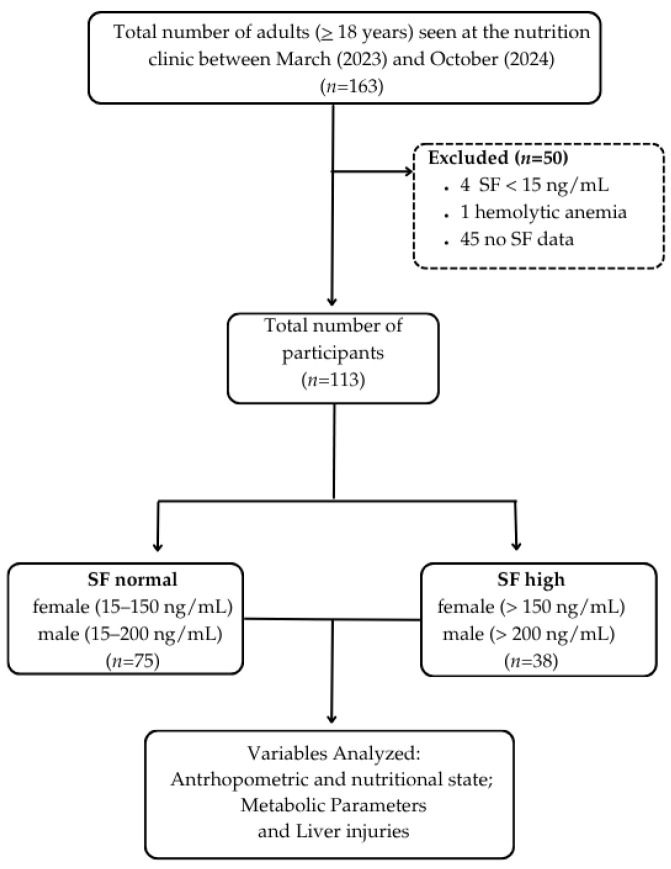
Flowchart of selection of study participants. SF (Serum Ferritin). Illustration created using Canva graphic software (freemium version), Canva Pty Ltd., Sydney, Australia.

From March 2023 to October 2024, the outpatient nutrition clinic attended 163 individuals, for whom socioeconomic, lifestyle, anthropometric, blood pressure, and dietary data were recorded, along with requested biochemical and metabolic tests, including SF levels and complete blood counts. SF levels were used to classify individuals as having normal SF (15–150 ng/mL for women and 15–200 ng/mL for men) or high SF (>150 ng/mL for women and >200 ng/mL for men), according to World Health Organization (WHO) recommendations [29]. Individuals with iron deficiency anemia (SF < 15 ng/mL), hemolytic anemia, or missing SF data were excluded. Pregnant women and individuals with viral hepatitis were also excluded from the study. The flowchart for participant selection is shown in Figure 1.

Socioeconomic Data: Age (years), sex (female and male), marital status, education level, and socioeconomic class were recorded. Family income was categorized according to the criteria of the Brazilian Association of Research Companies [30] as follows: AB (upper and upper-middle class: >R$5755.23 to 21,826.74 or >USD 1058.10 to 4012.85), C (middle class: >R$1965.87 to <R$5755.23 or >USD 361.42 to <1058.10), and DE (lower and lower-middle class: >R$900.60 to <R$1965.87 or >USD 165.57 to <361.42).

Lifestyle Data: Physical activity, smoking, and alcohol consumption were recorded. During the nutritional assessment, participants were asked about their physical activity, including type, duration (minutes), and frequency per week. Participants were classified as physically active if they engaged in at least 150–300 min per week of moderate-intensity, 75–150 min per week of vigorous-intensity aerobic activity, or an equivalent combination of moderate- and vigorous-intensity activity, according to WHO guidelines [31]. Alcohol consumption was assessed regarding type and weekly quantity (grams). The average alcohol content of common beverages was considered: beer = 5%, wine = 12%, and distilled spirits = 42% [32]. Alcohol intake was classified according to Cotrim et al. [33], with excessive consumption defined as >70 g/week for women and >140 g/week for men. Smoking status was self-reported. Individuals were considered smokers if they used cigarettes, cigars, cigarillos, pipes, hookahs, or electronic cigarettes, following the criteria of Malta et al. [34].

Anthropometry and Body Composition: Anthropometric measurements included body weight (kilograms, kg) and height (meters, m), performed according to the technique described by Gordon, Chumlea, and Roche [35]. Body mass index (BMI; kg/m^2^) was calculated from weight and height, and participants were classified according to WHO criteria [36] as underweight (BMI < 18.5 kg/m^2^), eutrophic (BMI 18.5–24.99 kg/m^2^), overweight (BMI 25–29.99 kg/m^2^), and obesity (BMI ≥ 30 kg/m^2^). Waist and hip circumferences (WC and HC, cm) were measured following Callaway [37], and waist-to-hip ratio (WHR) was calculated. WC and WHR were categorized according to WHO recommendations [36]. For women, WC < 80 cm and WHR < 0.85 were considered as no cardiovascular risk, while WC ≥ 80 cm and WHR ≥ 0.85 indicated cardiovascular risk. For men, WC < 94 cm and WHR < 1 indicated no cardiovascular risk, whereas WC ≥ 94 cm and WHR ≥ 1 indicated cardiovascular risk. Body composition was assessed using tetrapolar bioelectrical impedance (Biodynamics 450^®^, Biodynamics Corporation, Las Vegas, NV, USA), according to the manufacturer’s instructions [38]. Body fat percentage (BF%) was recorded and classified according to Lohman [39]: in females, BF% < 32% was considered acceptable and BF% ≥ 32% elevated; in males, BF% < 25% was considered acceptable and BF% ≥ 25% elevated. Lean mass percentage (LM%) was also measured.

Serum Biochemistry Analysis: Laboratory analyses were performed by the municipal clinical laboratory. After a 12-h fast, blood samples were collected, and serum was used to measure fasting blood glucose (mg/dL), total cholesterol (TC; mg/dL), triglycerides (TG; mg/dL), and high-density lipoprotein (HDL; mg/dL) using enzymatic colorimetric methods on the BIOPLUS-2000^®^ device (Bioplus Laboratory Products Ltd., Barueri, Brazil). Low-density lipoprotein (LDL; mg/dL) was calculated using the formula: LDL = TC − HDL − (TG/5) [40]. Glucose and triglyceride values were used to calculate the TyG index (ln[TG (mg/dL) × glucose (mg/dL)]/2), a marker of IR [41,42]. Uric acid (UA; mg/dL) was also measured using the enzymatic colorimetric method. SF (ng/mL) was assessed by chemiluminescence using the CENTAUR-SIEMENS^®^ system (Siemens Healthineers AG, Forchheim, Germany). Complete blood counts, including white blood cell and platelet profiles, were performed using the automated MINDRAY BC-5150^®^ device (Mindray Bio-Medical Electronics Co., Ltd., Shenzhen, China). Additionally, erythrocyte sedimentation rate (ESR) was determined according to the modified Westergren method [43], and C-reactive protein (CRP) was measured by immunoturbidimetric method using the BIOPLUS-2000^®^ device (Bioplus Laboratory Products Ltd., Barueri, Brazil). Classifications for serum biochemistry analyses are presented in Table 1.

Blood Pressure: Blood pressure was measured using an automatic device (G-Tech, BSP11^®^ (Accumed Medical and Hospital Products Ltd., Rio de Janeiro, Brazil) with participants seated, feet flat on the floor, left arm relaxed and supported at heart level, palm facing upwards, and bladder empty. Measurements were taken after participants had refrained from moderate or vigorous exercise, smoking, or alcohol consumption for at least 30 min previously, according to the protocol proposed by Barroso [32]. The classification of hypertension is presented in Table 1.

Metabolic Syndrome: The frequency of MS was assessed in females and males using the criteria established by the IDF [44]. MS was diagnosed when individuals presented elevated waist circumference (female: >80 cm; male: >90 cm) along with at least two of the following altered parameters: fasting glucose > 100 mg/dL or a previous diagnosis of diabetes; TG > 150 mg/dL; low HDL cholesterol (female: <50 mg/dL; male: <40 mg/dL) or use of lipid-lowering therapy; systolic blood pressure (SBP) > 130 mmHg and/or diastolic blood pressure (DBP) > 85 mmHg, or use of antihypertensive treatment.

Liver Analysis and Hepatic Pathology: Serum levels of hepatic enzymes, alanine aminotransferase (ALT) and aspartate aminotransferase (AST), were measured using a UV-optimized method on the BIOPLUS-2000^®^ device (Bioplus Laboratory Products Ltd., Barueri, Brazil). The Hepatic Steatosis Index (HSI) [48] was calculated using the formula: HSI = 8 × (ALT/AST ratio) + BMI (+2 if female; +2 if T2DM). Hepatic fibrosis was also assessed using the FIB-4 score, calculated as: FIB-4 = (age × AST)/(platelets × √ALT) [49]. The HSI and FIB-4 classifications are presented in Table 1.

Nutritional Assessment Data: Dietary intake was recorded using 24-h dietary recall (24HR). The reported amounts were converted to grams using the Table of Reported Measures for Food Consumed in Brazil, based on the 2008–2009 Family Budget Survey [50], or the Table for Evaluating Food Consumption in Household Measures [51]. Per capita consumption of oil, lard, and salt was also recorded. Nutrient intake, including energy, macronutrients (carbohydrates, lipids, and proteins), and micronutrients (vitamin C and iron), was calculated using Nutritional WebDiet^®^ software (https://webdiet.com.br/site/, A. M. Technological Solutions Ltd., São Luís, Brazil), which relies on the Brazilian Food Composition Table (TBCA) [52], and expressed in grams (g) and/or percentages (%). Intake of saturated fatty acids (SFA), monounsaturated fatty acids (MUFA), polyunsaturated fatty acids (PUFA), cholesterol, and fiber was also obtained for each participant. Iron bioavailability from the diet was estimated using the method of Monsen et al. [53], which classifies meals according to the absorption potential of iron, considering the content of heme iron (from meat) and vitamin C. Total iron, vitamin C, heme and non-heme iron, and absorption rates for heme (23%) and non-heme iron (3%, 5%, or 8%) were calculated for each meal and summed for daily intake. Additionally, the Food Frequency Questionnaire (FFQ) from the nutritional anamnesis was analyzed, categorizing food groups by daily, weekly, monthly, or rare/never consumption.

Statistical Analysis: In the present study, individuals were compared according to SF levels (normal vs. high), taking into account the nature of the variables (qualitative or quantitative) and the assumptions of each statistical test applied. The normality of quantitative variables was assessed using the Shapiro–Wilk test. For independent samples, the Student’s t-test (parametric) or the Mann–Whitney U test (non-parametric) was applied. For qualitative variables, the chi-square test (χ^2^) of independence with adjusted residual analysis was used (*p* > 1.96). When expected frequencies were below five, the Monte Carlo method with 5000 simulations was applied to obtain more robust *p*-values. Qualitative variables were expressed as absolute (n) and relative (%) frequencies, whereas quantitative variables were presented as median and interquartile range (Q1–Q3) for non-parametric data, or mean and standard deviation (SD) for parametric data. The significance level was set at 5% for all analyses. Variables with *p*-values < 0.20 in the initial analyses were considered eligible for inclusion in the subsequent multivariate analysis. Considering the sample size of the less frequent outcome event (*n* = 38) and to maintain an appropriate ratio between the number of events and predictors in the multivariate model, according to the standard recommendation of at least 10 events per independent variable, the final model was limited to a maximum of four variables. The final model included the following independent variables: sex, BF%, HSI (indicative of MASLD), and presence of MS. Due to the small number of cases in some categories and to avoid complete separation issues, model adjustment was performed using Firth’s penalized likelihood method, which corrects estimation bias in models with a small number of events. Individuals, classified according to SF levels (normal or high), were evaluated using multivariate statistical analysis. Variables were grouped as follows: Anthropometric data: BMI, overweight, WC, HC, WHR, BF%, LM%. Biochemical data: glucose, TG, TyG, RI, TC, HDL, LDL, AST, ALT, FIB-4, UA, CRP, ESR, HSI, and presence of MS. Dietary data: energy, carbohydrates, protein, lipids (SFA, MUFAs, PUFAs), cholesterol, fiber, total meat, total iron, total vitamin C, total heme iron, non-heme iron, and iron absorbed. Sociodemographic data: sex, age, marital status, education, income class, smoking, alcohol consumption, and physical activity. Individuals with incomplete data were excluded. For each group of variables, a dissimilarity matrix was constructed based on Gower distance (daisy function, cluster package). Differences between ferritin groups (normal vs. high) were assessed using permutational multivariate analysis of variance (PERMANOVA) via the adonis2 function in the vegan package. Results were visualized with Principal Coordinate Analysis (PCoA) plots using ggplot2, where each point represents an individual and the distance between points reflects calculated dissimilarity; closer points indicate greater similarity in the analyzed data (anthropometric, biochemical, etc.). Statistical analyses were performed using XLSTAT software (version 2019.2.2.59614, Addinsoft, Paris, France) within the Microsoft Excel 2016 environment, with the advanced statistical analysis module, and in R software (version 4.5.1).

## 3. Results

A total of 113 individuals participated in the study (Figure 1). Comparative sociodemographic characteristics between individuals with normal and high SF levels are presented in Table 2. Sex distribution differed significantly between groups (χ^2^ = 19.2; gl = 1; *p* < 0.0001), with a higher proportion of men in the high SF compared to the normal SF subjects. As such, a similar median age was found between the participant groups.

Marital status also differed between groups (χ^2^ = 10.9; gl = 3; *p* = 0.012). Thus, single status was predominant in the normal SF group, while married/civil union were more frequent in the high SF group. Similarly, the groups were different in relation to education level (χ^2^ = 15.9; gl = 6; *p* = 0.009), with a higher proportion of individuals who had completed high school in the high SF group, whereas incomplete higher education was more common in the normal SF group. However, family income, smoking, alcohol consumption, and physical activity did not differ significantly between normal and high SF subjects (Table 2).

Anthropometric and adiposity variables are presented in Table 3 for individuals with normal and high SF. BMI distribution differed between groups (χ^2^ = 13.1; gl = 3; *p* = 0.003). As such, eutrophic subjects were more prevalent in the normal SF group than in the high SF group. In contrast, we noted an increased prevalence of obesity in the high SF participants, compared to those participants in the normal SF group. As a consequence, the WC (χ^2^ = 8.4; gl = 1; *p* = 0.004) and BF% (χ^2^ = 9.2; gl = 1; *p* = 0.002) classifications were also different between groups. Thus, the SF group had a greater proportion of individuals with augmented WC and elevated BF%, compared to the normal SF group. In contrast, classifications for WHR and LM% did not differ between groups (Table 3).

Table 4 shows the classification of fasting biochemical variables, inflammation, hypertension and frequency of MS, when comparing groups with normal versus high SF. A greater number of individuals with high SF values had elevated fasting blood glucose (χ^2^ = 5.1; gl = 1; *p* = 0.023); TG (χ^2^ = 8.3; gl = 1; *p* = 0.003); IR (χ^2^ = 4.9; gl = 1; *p* = 0.027); and hypertension (χ^2^ = 9.6; gl = 1; *p* = 0.001), in relation to those with normal SF values. The median and respective SD values for each variable can be found in the Appendix A, Table A1.

Enzyme biomarkers, UA, and indices of MASLD and liver fibrosis were used to evaluate liver function in individuals with normal versus high SF (Table 5). Significant differences were observed between the groups with regard to values for UA (χ^2^ = 6.43; gl = 2; *p* = 0.016) and ALT (χ^2^ = 8.3; gl = 2; *p* = 0.008). Thus, subjects with elevated UA and ALT values were found in the SF high group, in comparison to normal SF subjects. The HSI, an indicator of MASLD, also differed significantly between groups (χ^2^ = 5.3; gl = 1; *p* = 0.022), with higher HSI values found in high SF individuals, compared to those with normal SF. In contrast, FIB-4 and AST did not differ between groups (Table 5).

The 24HR value was used to compare dietary intake in the normal and high SF subjects (Table 6). Only carbohydrate consumption differed significantly (U = 26; *p* = 0.024) between groups, with higher carbohydrate intake being observed in the high SF participants, compared to normal SF individuals. We also noted that, in the high SF group, there was also a trend towards higher fiber intake compared to the normal SF group (U = 24; *p* = 0.073). Energy, protein, lipids (including SFA, MUFAs, and PUFAs) cholesterol, and per capita oil and lard consumption did not differ between groups. Additionally, food parameters related to iron intake and bioavailability were similar between normal and high SF subjects (Table 6). Analysis of the participants’ FFQ revealed a significant difference between groups only for monthly sugar consumption, as shown in the Appendix A, Table A2.

The influence of variables on elevated SF levels was analyzed using odds ratios (OR) obtained from logistic regression (Table 7). Males exhibited a higher risk of elevated SF values (OR = 16.82), compared to females. Similarly, adiposity also influenced this risk, with individuals having increased BF% showing a higher likelihood of elevated SF (OR = 7.5). Other conditions, such as HSI and MS, did not significantly affect this risk (Table 7).

**Figure 2:**
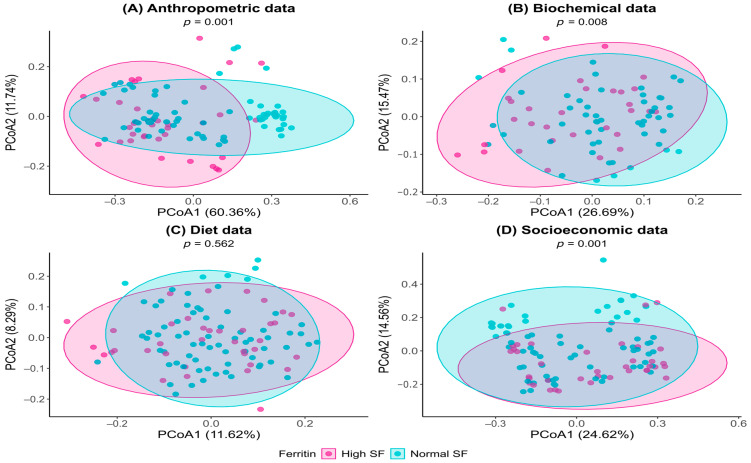
Principal Coordinate Analysis (PCoA) plots representing the dissimilarity between individuals with normal (blue) and high (pink) ferritin based on four groups of variables: (**A**) Anthropometric, (**B**) Biochemical, (**C**) Dietary, and (**D**) Socioeconomic. PERMANOVA *p*-values less than 0.05 indicate statistically significant differences between groups.

Finally, we clustered the anthropometric, biochemical, dietary, and socioeconomic data to perform a PCoA, comparing individuals with normal and high SF levels (Figure 2A–D). This analysis clearly demonstrated that normal and high SF individuals differ in terms of adiposity (Figure 2A; F_1,107_ = 12.8; *p* = 0.001), metabolism (Figure 2B; F_1,82_ = 3.79; *p* = 0.008), and sociodemographic characteristics (Figure 2D; F_1,110_ = 6.53; *p* = 0.001).

According to PCoA, anthropometric data explained 60.36% of the variation, with BMI, WC, and BF% classification being the primary contributing variables. As a result, subjects in the high SF group exhibited higher adiposity, reflected in elevated values for these measurements. Biochemical and liver injury indices (Figure 2B) accounted for 26.69% of the total variation on PCoA, primarily influenced by TyG, TG, and HSI. Elevated values of these variables indicated a profile associated with IR and MASLD. Additionally, the presence of MS also contributed to PCoA, with high SF individuals showing an increased risk of MS. Interestingly, sociodemographic data (Figure 2D) accounted for 24.62% of the variability on PCoA and was primarily influenced by physical activity. In this regard, individuals with normal SF values were more physically active than those with high SF levels. On the other hand, these differences did not appear to be related to dietary profile, as shown in Figure 2C (F_1,106_ = 0.89; *p* = 0.562).

## 4. Discussion

Altered SF is an important clinical biomarker for evaluating iron status and is especially useful for detecting iron deficiency [8]. However, growing evidence has shown that SF can also indicate metabolic abnormalities, such as those associated with obesity, diabetes, and liver diseases, including MASLD [7,54,55]. Circulating SF levels are influenced by sex, age, adiposity, inflammation, and race [1,11,56,57]. Therefore, it is important to characterize the SF profile of a population to determine its potential in identifying metabolic abnormalities and to better tailor dietary and therapeutic interventions aimed at preventing chronic disease over the lifespan [55,58]. To the best of our knowledge, this is the first study conducted with subjects living in southern Brazil, categorized according to normal and high SF levels, comparing their anthropometric, metabolic, dietary, and liver function parameters.

In our sample, individuals in the high SF group had lower educational levels, were more frequently in a civil union or married, and were predominantly male. It has been shown that socioeconomic status modulates iron homeostasis [59,60,61]. Moreover, we also demonstrated that men had a higher risk of elevated SF values, compared to women. Our results are consistent with a study conducted in southern Brazil, which reported that hyperferritinemia is common in Gaucher disease, particularly in untreated, older male individuals with higher BMI [62].

A prospective cohort study including more than 13,000 middle-aged Korean men showed that baseline elevation of SF levels was positively and significantly associated with the development of MS during a 5-year follow-up [63]. According to Park et al. [63], elevated SF values were significantly higher, or tended to be higher, in men who met the TG criterion for MS, either resolved or developed, over the follow-up period. In agreement with these findings, in our sample, subjects in the high SF group exhibited a higher prevalence of altered TG levels compared to individuals with normal SF.

Obesity has been associated with elevated circulating SF levels and worse metabolic status [12,14]. Consistent with this, our results showed that individuals in the high SF group had a higher frequency of obesity and a greater percentage of visceral adiposity, as indicated by elevated WC and BF%. Similar findings were reported in the European Prospective Investigation into Cancer and Nutrition (EPIC) study, which analyzed iron and SF levels in 828 individuals from seven of the ten participating countries (Germany, Spain, France, Italy, the United Kingdom, Sweden, and the Netherlands) [64].

In a large population-based Chinese study, income, smoking, and alcohol consumption were associated with altered circulating SF levels, particularly in men [65]. However, in the present study, we did not find significant differences in these variables when comparing normal and high SF subjects.

Augmented SF has been associated with metabolic dysfunction, characterizing a so-called metabolic hyperferritinaemia. Consequently, altered SF levels are associated with disruptions in glycemic and lipid control, primarily attributed to IR [11]. Accordingly, our data also demonstrated that participants in the high SF group more frequently exhibited altered fasting blood glucose, TG, and IR (evaluated by the TyG index), as well as hypertension, resulting in a higher prevalence of MS in this group. These findings are consistent with a meta-analysis showing that SF is positively associated with MS, with high TG and glucose being the components most strongly linked to SF [66]. It is important to note that the high SF group had increased visceral adiposity, which is closely related to glucose intolerance and dyslipidemia [14]. Augmented visceral adiposity and the associated pro-inflammatory state have been linked to disrupted iron homeostasis, primarily because elevated IL-6 levels promote increased liver hepcidin production, which leads to intracellular iron overload [1]. Moreover, a negative relationship between elevated SF and impaired β-cell function also has been reported. In this regard, hyperferritinemia can lead to reduced insulin secretion and provoke β-cell ferroptosis, a novel mechanism of cell death that is induced by iron overload [7,67,68]. Therefore, evaluating SF levels and their association with MS is critical for preventing MASH and its deleterious hepatic consequences and the in-depth analysis of ferroptosis and its potential molecular mechanisms in human diseases may offer additional strategies for clinical prevention and treatment [25].

The liver is a central organ for iron, glucose, and lipid homeostasis, and all these processes are negatively affected by obesity and IR [26,69]. In the present study, we evaluated enzymatic biomarkers and hepatic indices to determine whether SF levels could be associated with liver injury. Our results showed alterations in liver disease biomarkers, such as ALT and MASLD, in individuals of the high SF group. In MASLD, UA is considered a marker of oxidative stress and a pathogenic factor associated with MS and CVD [70]. Consistently, in the present study, a higher frequency of elevated UA was observed in the high SF group. This finding aligns with a population-based study including healthy subjects of both sexes from the National Health and Nutrition Examination Survey III, which demonstrated a positive correlation between UA and SF [71]. Serum UA is increasingly recognized as a determinant of fatty liver disease due to its association with metabolic disorders [72,73]. Elevated serum UA is also linked to inflammation, IR, and atherosclerosis [74]. ALT is an enzyme that is predominantly found in the liver, and elevated serum ALT levels indicate liver injury from various causes, including iron deposition [75]. In our study, we observed a higher frequency of ALT alterations in the high SF group. Similarly, He et al. [76] reported that elevated SF levels were positively associated with ALT and MASLD.

In the present work, individuals in the high SF group exhibited elevated HSI values, which is a valuable biomarker for MASLD [77]. Similarly, Shah and Kowdley [78] reported that participants in the highest quartile of SF levels were more likely to have MASLD based on HSI. A follow-up study showed that hyperferritinemia was associated with a 50% increased risk of liver-related events and a 27% increase in all-cause mortality. These authors also demonstrated that serum SF improved the predictive performance for liver-related events and mortality, when combined with non-invasive scoring systems, such as FIB-4 [79]. In contrast, we did not find differences in the FIB-4 index in our study, suggesting that the participants had a milder degree of liver injury. Importantly, studies with liver biopsy specimens have shown that a mixed pattern of hepatic iron deposition is associated with the presence of MASH, while SF levels increase with worsening fibrosis up to a pre-cirrhotic stage, but are not independent predictors of advanced fibrosis [80].

Hepatocyte iron overload is involved in several liver-associated diseases [81,82]. However, hyperferritinemia may or may not be associated with increased hepatic iron in MASLD individuals. A recent consensus statement indicates that not all patients with metabolic dysfunction or fatty liver present increased SF levels. According to these authors, patients with metabolic hyperferritinemia constitute a subset with distinct risk factors, pathophysiology and clinical outcomes, warranting special attention [11]. These findings suggest that the liver abnormalities observed in our high SF participants could be related to obesity and its associated comorbidities. Elevated pro-inflammatory cytokines, such as TNF-α, IL-1β, and IL-6, upregulate ferritin synthesis. Moreover, SF levels have been associated with the degree of hepatic fat accumulation and the severity of liver damage, as recently reviewed by Shen et al. [82]. Oxidative stress, resulting from the accumulation of harmful intermediates of fatty acids due to incomplete intracellular oxidation (lipotoxicity), may directly enhance ferritin transcription [11,54,82]. It is important to highlight that a previous diagnosis of hereditary hemachromatosis or transferrin saturation (> 50%) are exclusion criteria for metabolic hyperferritinemia [11].

Several factors associated with obesity can influence diet and nutrient absorption, including iron and SF [1,83]. Therefore, in the present study, we applied 24HRs, a well-recognized tool for analyzing dietary intake and estimating iron consumption [53]. In our sample, participants in the normal and high SF groups had similar iron intake and absorption. For adult men and women (aged 18–50 years), the recommended daily intake of iron is 8 mg and 18 mg, respectively [84]. Dietary iron is present in two forms, heme and non-heme iron, which differ in bioavailability, absorption, and dietary sources [85]. Average daily iron intake is 10–15 mg in humans, of which only 1–2 mg is absorbed through the intestinal system [86]. Furthermore, in healthy young women, heme iron has been identified as an independent predictor of SF, suggesting that the consumption of foods such as meat (beef, veal, lamb, poultry, and fish) contributes to maintaining adequate SF levels [87]. In our study, none of these dietary nutrients differed between groups.

Ascorbic acid (vitamin C) is the most efficient enhancer of non-heme iron absorption and must be obtained from the diet [88]. Some studies have suggested that iron absorption may be influenced by total caloric intake, as more calorically dense meals may contain a higher proportion of iron absorption inhibitors, such as calcium and phytates, and a lower proportion of enhancers, such as vitamin C, potentially reducing non-heme iron bioavailability [86]. However, this parameter did not differ between groups in our study.

The role of dietary iron in obesity remains controversial, as highlighted by a recent review [89]. As previously mentioned, pro-inflammatory processes associated with obesity increase hepcidin, a protein that regulates intestinal iron absorption [1]. Additionally, excessive caloric intake may induce intestinal inflammation and reduce iron absorption [90]. In this context, we found that high SF subjects consumed more carbohydrates compared to individuals with normal SF. The relationship between carbohydrate intake and increased SF is not yet well established. In athletes, carbohydrate intake has been shown to modulate iron levels without affecting SF [91]. Furthermore, energy-deficient diets or low carbohydrate availability may increase hepcidin in the absence of inflammation and elevate circulating iron and SF levels [92]. However, here, neither OR nor PCoA analysis indicated significant effects of diet on high SF values. Therefore, it is unlikely that the higher carbohydrate consumption observed in high SF individuals contributed to their elevated SF levels.

On the other hand, altered glycemia, TG, and IR, especially when associated with elevated adiposity, are clearly linked to hyperferritinemia [93], particularly in men. Men and women differ in adipose tissue distribution: men tend to accumulate more visceral fat, resulting in the classic android body shape, which is highly correlated with increased cardiovascular risk. Conversely, women accumulate more fat in the subcutaneous compartment before menopause, a characteristic that provides protection against the negative consequences associated with obesity and MS. After menopause, however, fat deposition shifts toward the visceral compartment [94]. It is important to note that, in our study, the median ages were 31 years (23–41.5) in the normal SF group and 41 years (31.2–49.8) in the high SF group, suggesting an age range unlikely to be significantly influenced by menopause, which occurs on average between 48 and 50 years in women [95].

In the present study, individuals with high SF clustered multiple abnormalities, including obesity, hyperglycemia, IR, and liver injury, predominantly in men. This pattern is clearly highlighted by the PCoA analysis, which showed that high SF individuals differed in terms of adiposity, metabolism, and physical activity, without differences in dietary intake, compared to normal SF subjects.

Regarding the limitations of our study, we note the small sample size, which may influence statistical analyses, particularly logistic regression. To address this, we applied the PCoA method, which is useful for reconciling multivariate data and establishing relationships based on similarities or dissimilarities. This approach is particularly advantageous when dealing with complex relationships among multiple variables, as in our dataset. Additionally, 24HR analysis has limitations, especially for assessing micronutrient intake, as noted by Raina [96]. Nevertheless, dietary data collection was of secondary importance in this study, and 24HR is a method widely used in clinical practice. Measures of serum IL-6, serum iron, and transferrin would be valuable to further consolidate our findings. Finally, here we adopted a cross-sectional design analyzing data from a single outpatient nutrition clinic. This design demonstrates high internal validity, providing consistent results for the sample analyzed, but as indicated for Wang and Cheng [97] it limited external validity, thereby restricting the generalizability of the findings to other populations. The cross-sectional nature of the study also limits the ability to establish directionality or causality between variables, since the simultaneity of measurements prevents the temporal evaluation of events. Despite these limitations, our results are consistent with other studies that used larger sample sizes [79] and more specific clinical and molecular biomarkers [98,99].

## 5. Conclusions

In conclusion, individuals with elevated SF levels are predominantly male, obese, exhibiting greater visceral fat deposition, metabolic abnormalities and IR. Which are associated with a higher prevalence of MS and liver injury. These results suggest that SF, along with other iron-related biomarkers, may have potential clinical utility as indicators of obesity-related conditions, particularly liver dysfunction, such as MASLD. However, it is important to emphasize that these findings reflect associations rather than causal relationships, and prospective studies are needed to confirm their clinical relevance.

## Figures and Tables

**Table 1:** Serum biochemistry analyses classifications.

Serum Biochemistry	Classification	Reference
SF	Normal (15 a 150 ng/mL for female and 15 a 200 ng/mL for male) High SF (>150 ng/mL for female and >200 ng/mL for male).	WHO [29].
Glucose	Normal (<100 mg/dL); High (>100 mg/dL), for both sexes.	International Diabetes Federation (IDF) [44].
TG	Normal (<150 mg/dL); High (>150 mg/dL), for both sexes.	IDF [44].
TC	Normal (<190 mg/dL); High (>190 mg/dL), for both sexes.	Précoma et al. [45].
HDL	Normal (female > 50 mg/dL and male > 40 mg/dL); Decreased (<50 mg/dL female and <40 mg/dL male).	IDF [44].
LDL	Normal (<130 mg/dL); High (>130 mg/dL), for both sexes.	Précoma et al. [45].
TyG	>4.55 to female and >4.68 to male were indicative of IR.	Guerrero-Romero et al. [41].
ESR	Normal (female < 20 mm and High > 20 mm); Normal (male < 10 mm and High > 10 mm).	Wetteland et al. [46].
CRP	<5mg/dL (Negative); >5 mg/dL (Positive).	Khedr et al. [47].
UA	Decreased (<1.5 mg/dL); Normal (1.5 to 6 mg/dL); High (>6 mg/dL), for both sexes.	Laboratory
Hypertension	No (SBP: <130 mmHg and DBP < 85 mmHg); Yes (SBP: >130 mmHg and DBP > 85 mmHg) or in hypertension treatment, for both sexes.	IDF [44].
AST and ALT	Decreased (<10 U/L); Normal (10 a 37 U/L); High (>37 U/L), for both sexes.	Laboratory.
HSI	Yes (>36) No (<36)	Lee et al. [48].
FIB-4	No risk of fibrosis (score < 1.3); Risk of fibrosis (>1.3), for both sexes.	Shah et al. [49].

**Table 2:** Distribution of socioeconomic and lifestyle characteristics of participants according to SF classification (normal vs. high). Brazil, 2023–2024.

Variable	Category	Normal SF *n* (%)	High SF *n* (%)	*p*-Value
Sex	Female	67 (89.3%) #	20 (52.6%)	**<0.0001 ^a^**
	Male	8 (10.7%)	18 (47.4%) #	
Age	-	31 (23–41.5)	41 (31.2–49.8)	0.315 ^c^
Marital Status	Single	40 (53.3%) #	8 (21.1%)	**0.012 ^b^**
	Married/Civil Union	28 (37.4%)	25 (65.7%) #	
	Divorced	4 (5.3%)	3 (7.9%)	
	Widower	3 (4%)	2 (5.3%)	
Education	Incomplete Elementary Education	6 (8%)	6 (15.8%)	**0.009 ^b^**
	Complete Elementary Education	1 (1.3%)	0 (0%)	
	Incomplete High School	2 (2.7%)	1 (2.6%)	
	Complete High School	15 (20%)	16 (42.1%) #	
	Incomplete Higher Education	27 (36%)#	2 (5.3%)	
	Complete Higher Education	19 (25.3%)	11 (28.9%)	
	Posgraduate	5 (6.7%)	2 (5.3%)	
Family Income Class	AB	14 (26.3%)	10 (18.9%)	0.387 ^a^
	C	43 (60.5%)	23 (58.1%)	
	DE	17 (13.2%)	5 (23%)	
Smoker	No	69 (92%)	35 (92.1%)	0.984 ^a^
	Yes	6 (8%)	3 (7.9%)	
Alcohol Consumption	No	41 (54.7%)	20 (52.6%)	0.838 ^a^
	Yes	34 (45.3%)	18 (47.4%)	
Classification of Alcohol Consumption	Not Excessive	68 (90.7%)	37 (97.4%)	0.189 ^a^
	Excessive	7 (9.3%)	1 (2.6%)	
Physical Activity	No	27 (36%)	16 (42.1%)	0.528 ^a^
	Yes	48 (64%)	22 (57.9%)	
Minutes per week	-	150 (0–300)	120 (0–281.2)	0.661 ^c^
Active	No	36 (48%)	22 (57.9%)	0.320 ^a^
	Yes	39 (52%)	16 (42.1%)	

Note: AB (upper and upper-middle class: >R$5755.23 to 21,826.74 or >USD 1058.10 to 4012.85); C (middle class: >R$1965.87 to <R$5755.23 or >USD 361.42 to <1058.10); DE (lower and lower-middle class: >R$900.60 to <R$1965.87 or >USD 165.57 to <361.42). Age and Minutes per week: Median (Q1–Q3). ^a^ χ^2^ test for independence. ^b^ Monte Carlo method. ^c^ Mann–Whitney-U test. Bold values indicate statistically significant contribution (*p* < 0.05). # represents category with significant difference by adjusted residuals (*p* > 1.96).

**Table 3:** Distribution of participants’ anthropometric parameters, according to SF classification (normal vs. high). Brazil, 2023–2024.

Variable	Category	Normal SF *n* (%)	High SF *n* (%)	*p*-Value
BMI	Underweight	3 (4%)	0 (0%)	**0.003 ^b^**
	Eutrophic	31 (41.3%) #	5 (13.2%)	
	Overweight	20 (26.7%)	12 (31.6%)	
	Obesity	21 (28%)	21 (55.3%) #	
WC	No Risk	37 (49.3%) #	8 (21.1%)	**0.004 ^a^**
	With Risk	38 (50.7%)	30 (78.9%) #	
WHR	No Risk	57 (77%)	28 (73.7%)	0.874 ^a^
	With Risk	17 (23%)	10 (26.3%)	
BF%	Acceptable	40 (54.8%) #	9 (24.3%)	**0.002 ^a^**
	High	33 (45.2%)	28 (75.7%) #	
LM%	-	70 ± 8.1	68.4 ± 6.8	0.301 ^c^

Note: Mean ± SD. ^a^ χ^2^ test for independence. ^b^ Monte Carlo method. ^c^ Student’s *t*-test. *p*-value. Bold values indicate statistically significant contribution (*p* < 0.05). # represent categories with significant differences based on adjusted residuals (*p* > 1.96).

**Table 4:** Distribution of biochemical parameters, insulin resistance, hypertension and presence of MS in participants, according to the SF classification (normal vs. high). Brazil, 2023–2024.

Variable	Category	Normal SF *n* (%)	High SF *n* (%)	*p*-Value
Glucose	Normal	67 (91.8%) #	29 (76.3%)	**0.023 ^a^**
	High	6 (8.2%)	9 (23.7%) #	
TG	Normal	65 (86.7%) #	24 (63.2%)	**0.003 ^a^**
	High	10 (13.3%)	14 (36.8%) #	
TC	Normal	54 (70.1%)	21 (58.3%)	0.216 ^a^
	High	23 (29.9%)	15 (41.7%)	
HDL	Normal	13 (17.3%)	9 (23.7%)	0.420 ^a^
	Decreased	62 (82.7%)	29 (76.3%)	
LDL	Normal	58 (77.3%)	28 (73.7%)	0.667 ^a^
	High	17 (22.7%)	10 (26.3%)	
IR	No	52 (71.2%) #	19 (50%)	**0.027 ^a^**
	Yes	21 (28.8%)	19 (50%) #	
CRP	Negative	40 (66.7%)	24 (68.6%)	0.849 ^a^
	Positive	20 (33.3%)	11 (31.4%)	
ESR	Normal	31 (53.4%)	20 (57.1%)	0.729 ^a^
	High	27 (46.6%)	15 (42.9%)	
Hypertension	Yes	16 (21.3%)	19 (50%) #	**0.001 ^a^**
	No	59 (78.7%) #	19 (50%)	
MS	No	59 (78.7%) #	18 (47.4%)	**0.001 ^a^**
	Yes	16 (21.3%)	20 (52.6%) #	

Note: ^a^ χ^2^ test for independence. Bold values indicate statistically significant contribution (*p* < 0.05). # represent categories with significant differences based on adjusted residuals (*p* > 1.96).

**Table 5:** Distribution of participants’ liver parameters according to SF classification (normal vs. high). Brazil, 2023–2024.

Variable	Category	Normal SF *n* (%)	High SF *n* (%)	*p*-Value
UA	Decreased	1 (1.5%)	0 (0%)	**0.016 ^b^**
	Normal	60 (88.2%) #	27 (71%)	
	High	7 (10.3%)	11 (29%) #	
AST	Normal	58 (82.8%)	27 (73%)	0.228 ^a^
	High	12 (17.2%)	10 (27%)	
ALT	Decreased	1 (1.4%)	0 (0%)	**0.008 ^b^**
	Normal	66 (94.3%) #	29 (78.4%)	
	High	3 (4.3%)	8 (21.6%) #	
FIB4	No Risk fibrosis	56 (83.6%)	30 (83.3%)	0.974 ^a^
	With Risk fibrosis	11 (16.4%)	6 (16.7%)	
HSI > 36	No	39 (55.7%) #	12 (32.4%)	**0.022 ^a^**
	Yes	31 (44.3%)	25 (67.6%) #	

Note: ^a^ χ^2^ test for independence. ^b^ Monte Carlo method. Bold values indicate statistically significant contribution (*p* < 0.05). # represent categories with significant differences based on adjusted residuals (*p* > 1.96).

**Table 6:** Distribution of participants’ 24HR dietary data, according to SF classification (normal vs. high). Brazil, 2023–2024.

Variable	Normal SF Median (Q1–Q3)	High SF Median (Q1–Q3)	*p*-Value
Energy (kcal)	1692 (1315–1995)	1718 (1076.2–2411.2)	0.412 ^a^
Carbohydrate (g)	189.5 (146.7–278.5)	212.2 (129.9–287.1)	**0.024 ^a^**
Protein (g)	68.6 (52.3–97.2)	79.5 (60.7–107)	0.788 ^a^
Lipids (g)	55.7 (40–74.8)	64.3 (36–88.1)	0.164 ^a^
SFA (g)	17.8 (11.8–25)	18.2 (12.6–28)	0.315 ^a^
MUFA (g)	15.6 (11.2–22.9)	18.4 (11.4–24.7)	0.412 ^a^
PUFA (g)	12.3 (9.2–17.6)	12.9 (9.3–18.4)	0.927 ^a^
Cholesterol (mg)	261.9 (167.8–426.9)	270.1 (178.3–477.3)	0.527 ^a^
Fibers (g)	18.3 (10.9–25.4)	22.7 (15.8–30.4)	0.073 ^a^
Per capita oil (ml)	10 (6.6–15)	10 (6.1–15)	0.970 ^a^
Per capita lard (g)	5.5 (2.6–14.7)	5.6 (4.2–8.3)	0.558 ^a^
Total vitamin C (mg)	49.2 (20.4–119)	57.1 (25.5–137.3)	0.648 ^a^
Total meat (g)	120 (60–217.5)	150 (100.5–200)	0.927 ^a^
Total iron (mg)	10.9 (7.5–14.4)	10.1 (6.5–13.1)	0.164 ^a^
Total heme iron (mg)	0.9 (0.3–1.8)	0.8 (0.3–1.9)	0.788 ^a^
Total non-heme iron (mg)	9.3 (6.8–12.5)	9.2 (5.6–12.6)	0.164 ^a^
Total iron absorbed (mg)	0.7 (0.5–1.1)	0.6 (0.5–0.9)	0.315 ^a^

Note: ^a^ Mann–Whitney-U test. Bold values indicate statistically significant contribution (*p* < 0.05).

**Table 7:** Logistic regression model parameters. Brazil, 2023–2024.

Source	Value	Pr > χ^2^	OR [IC 95%] *
Intercept	−2.46	<0.0001	
Sex-Female	0.00		
Sex-Male	2.82	**<0.0001**	16.82 [4.48–63.1]
BF%-Acceptable	0.00		
BF%-Elevated	2.02	**0.004**	7.5 [1.94–29.03]
HSI < 36-No	0.00		
HSI > 36-Yes	−0.43	0.47	0.65 [0.20–2.11]
MS-No	0.00		
MS-Yes	0.55	0.29	1.74 [0.62–4.84]

Note: * OR = Odds Ratio. * 95%CI = Lower limit and upper limit of the 95% confidence interval. Bold values indicate statistically significant contribution (*p* < 0.05).

## Data Availability

The original contributions presented in this study are included in the article. Further inquiries can be directed to the corresponding author.

## Abbreviations

The following abbreviations are used in this manuscript:

SF	Serum ferritin
TG	Tryglicerídes
TC	Total cholesterol
HDL	High density lipoprotein
LDL	Low density lipoprotein
IR	Insulin resistance
TyG	Glucose and triglycerides index
ESR	Erythrocyte sedimentation rate
CRP	C-reactive protein
UA	Uric acid
SBP	Systolic blood pressure
DPB	Diastolic blood pressure
ALT	Alanine transaminase
AST	Aspartate transaminase
HIS	Hepatic steatosis index
FIB-4	Hepatic fibrose factor score
MS	Metabolic syndrome
BMI	Body mass index
WC	Waist circumference
WHR	Waist-to-hip ratio
BF	Body fat
LM	Lean mass
24HR	24-h diet recall
Kcal	kilocalories
g	Grams
SFA	Saturated fatty acid
MUFA	Monounsaturated fatty acid
PUFA	Polyunsaturated fatty acid
mg	Milligrams
ml	Milliliter

## Appendix A

Supplementary Data

**Table A1:** Distribution of participants’ biochemical parameters according to SF classification (normal vs. high). Brazil, 2023–2024.

Variable	Category	Normal SF Median (Q1–Q3)	High SF Median (Q1–Q3)	*p*-Value
SBP (mmHg)	-	117.5 (107.2–127)	132 (113.2–140.2)	0.952 ^b^
DBP (mmHg)	-	74.5 (68.2–80.8)	81 (68.5–86.8)	0.394 ^b^
Glucose (mg/dL)	-	77 (72–89)	87 (80.2–97)	0.127 ^b^
TG (mg/dL)	-	86 (61.5–117)	114.5 (88.2–190.2)	0.788 ^b^
TyG	-	4.4 (4.2–4.6)	4.6 (4.5–4.9)	0.927 ^b^
TC (mg/dL)	-	170.8 (147–192.5)	185 (156.2–203)	0.788 ^b^
HDL (mg/dL)	-	45 (41–48)	40 (38–44.8)	0.909 ^b^
LDL (mg/dL)	-	105.4 (81.9–124.7)	105.7 (90.2–128.2)	0.927 ^b^
AST (U/L)	-	27 (21–34)	29 (22–38)	0.679 ^b^
ALT (U/L)	-	20 (15–25.8)	26 (20–35.6)	0.630 ^b^
FIB4	-	0.7 (0.5–1.2)	0.9 (0.7–1.1)	0.315 ^b^
UA (mg/dL)	-	4.2 (3.2–4.9)	4.8 (3.6–6.1)	0.648 ^b^
ESR (mm)	-	15 (10–24.8)	12 (10–16)	0.206 ^b^
Platelets (mil/mm^3^)	-	235 (209–283)	249 (221–266)	0.436 ^b^
Leukocytes (mm^3^)	-	6400 (5550–7800)	6400 (5599.8–8950)	0.788 ^b^
Rod neutrophils (mm^3^)	-	57 (40–72)	61 (0–75)	0.836 ^b^
Eosinophils(mm^3^)	-	144 (104–226.5)	137 (69.2–191.2)	0.648 ^b^
Segmented neutrophils (mm^3^)	-	3510 (3000–4298)	3650 (2669–4929)	0.788 ^b^
Monocytes (mm^3^)	-	300 (220–388)	312 (252–456)	0.527 ^b^
Lymphocytes (mm^3^)	-	2280 (1912–2668.5)	2171.5 (2018–2606.8)	0.927 ^b^
HSI	-	34.2 (30.9–38.6)	39.9 (35.5–45.4)	0.788 ^b^

Note: ^b^ Mann–Whitney U test.

**Table A2:** Distribution of food groups from the participants’ Food Frequency Questionnaire (FFQ) according to the SF classification (normal vs. high). Brazil. 2023–2024.

Variable	Category	Normal SF *n* (%)	High SF *n* (%)	*p*-Value
Cookies, cakes	Never or rarely	15 (20.8%)	7 (18.4%)	0.744 ^a^
	Monthly	12 (16.7%)	4 (10.5%)	
	Weekly	34 (47.2%)	19 (50%)	
	Daily	11 (15.3%)	8 (21.1%)	
Masses	Never or rarely	5 (6.9%)	4 (10.5%)	0.534 ^b^
	Monthly	9 (12.5%)	5 (13.2%)	
	Weekly	55 (76.4%)	25 (65.8%)	
	Daily	3 (4.2%)	4 (10.5%)	
Whole grains	Never or rarely	32 (44.4%)	13 (34.2%)	0.257 ^b^
	Monthly	3 (4.2%)	3 (7.9%)	
	Weekly	26 (36.1%)	11 (28.9%)	
	Daily	11 (15.3%)	11 (28.9%)	
Candy	Never or rarely	12 (16.7%)	6 (16.2%)	0.091 ^b^
	Monthly	5 (6.9%)	6 (16.2%)	
	Weekly	31 (43.1%)	20 (54.1%)	
	Daily	24 (33.3%)	5 (13.5%)	
Butter, bacon, lard, lard	Never or rarely	29 (40.8%)	11 (29.7%)	0.212 ^b^
	Monthly	3 (4.2%)	3 (8.1%)	
	Weekly	17 (23.9%)	15 (40.5%)	
	Daily	22 (31%)	8 (21.6%)	
Margarine, mayonnaise	Never or rarely	34 (47.2%)	14 (37.8%)	0.428 ^b^
	Monthly	3 (4.2%)	0 (0%)	
	Weekly	20 (27.8%)	13 (35.1%)	
	Daily	15 (20.8%)	10 (27%)	
Snacks	Never or rarely	13 (18.1%)	7 (18.9%)	0.426 ^b^
	Monthly	29 (40.3%)	9 (24.3%)	
	Weekly	28 (38.9%)	19 (51.4%)	
	Daily	2 (2.8%)	2 (5.4%)	
Preserved food	Never or rarely	46 (63.9%)	21 (56.8%)	0.8 ^b^
	Monthly	13 (18.1%)	6 (16.2%)	
	Weekly	12 (16.7%)	9 (24.3%)	
	Daily	1 (1.4%)	1 (2.7%)	
Fried foods	Never or rarely	28 (38.9%)	11 (29.7%)	0.675 ^b^
	Monthly	10 (13.9%)	8 (21.6%)	
	Weekly	29 (40.3%)	16 (43.2%)	
	Daily	5 (6.9%)	2 (5.4%)	
Vegetables	Never or rarely	4 (5.6%)	2 (5.4%)	0.663 ^b^
	Monthly	1 (1.4%)	1 (2.7%)	
	Weekly	25 (34.7%)	17 (45.9%)	
	Daily	42 (58.3%)	17 (45.9%)	
Beans	Never or rarely	3 (4.2%)	0 (0%)	0.287 ^b^
	Monthly	2 (2.8%)	3 (7.9%)	
	Weekly	23 (31.9%)	15 (39.5%)	
	Daily	44 (61.1%)	20 (52.6%)	
Leafy greens	Never or rarely	3 (4.2%)	2 (5.4%)	1 ^b^
	Monthly	1 (1.4%)	0 (0%)	
	Weekly	20 (27.8%)	11 (29.7%)	
	Daily	48 (66.7%)	24 (64.9%)	
Tubers	Never or rarely	3 (4.2%)	1 (2.7%)	0.702 ^b^
	Monthly	2 (2.8%)	2 (5.4%)	
	Weekly	55 (76.4%)	25 (67.6%)	
	Daily	12 (16.7%)	9 (24.3%)	
Fruits	Never or rarely	1 (1.4%)	3 (8.1%)	0.376 ^b^
	Monthly	1 (1.4%)	1 (2.7%)	
	Weekly	21 (29.2%)	9 (24.3%)	
	Daily	49 (68.1%)	24 (64.9%)	
Soft drinks, juices	Never or rarely	28 (38.9%)	18 (48.6%)	0.557 ^b^
	Monthly	6 (8.3%)	1 (2.7%)	
	Weekly	30 (41.7%)	13 (35.1%)	
	Daily	8 (11.1%)	5 (13.5%)	
Sweetener	Never or rarely	66 (91.7%)	33 (89.2%)	1 ^b^
	Weekly	1 (1.4%)	1 (2.7%)	
	Daily	5 (6.9%)	3 (8.1%)	
Diet e light	Never or rarely	69 (95.8%)	31 (83.8%)	0.073 ^b^
	Monthly	2 (2.8%)	2 (5.4%)	
	Weekly	1 (1.4%)	2 (5.4%)	
	Daily	0 (0%)	2 (5.4%)	
Ready-made seasonings	Never or rarely	53 (73.6%)	28 (75.7%)	0.681 ^b^
	Monthly	1 (1.4%)	2 (5.4%)	
	Weekly	6 (8.3%)	3 (8.1%)	
	Daily	12 (16.7%)	4 (10.8%)	
Sugar	Never or rarely	21 (29.2%)	15 (40.5%)	**0.024 ^b^**
	Monthly	0 (0%)	3 (8.1%) #	
	Weekly	18 (25%)	4 (10.8%)	
	Daily	33 (45.8%)	15 (40.5%)	
Oilseeds	Never or rarely	36 (50%)	16 (43.2%)	0.857 ^a^
	Monthly	9 (12.5%)	6 (16.2%)	
	Weekly	16 (22.2%)	10 (27%)	
	Daily	11 (15.3%)	5 (13.5%)	

Note: ^a^ χ^2^ test for independence. ^b^ Monte Carlo method. Bold values indicate statistically significant contribution (*p* < 0.05). # represent categories with significant differences based on adjusted residuals (*p* > 1.96).
